# Clobetasol Modulates Adult Neural Stem Cell Growth via Canonical Hedgehog Pathway Activation

**DOI:** 10.3390/ijms20081991

**Published:** 2019-04-23

**Authors:** Nunzio Vicario, Joshua D. Bernstock, Federica M. Spitale, Cesarina Giallongo, Maria A.S. Giunta, Giovanni Li Volti, Massimo Gulisano, Giampiero Leanza, Daniele Tibullo, Rosalba Parenti, Rosario Gulino

**Affiliations:** 1 Lab of Cellular and Molecular Physiology, Department of Biomedical and Biotechnological Sciences, Section of Physiology, University of Catania, 95123 Catania, Italy; nunziovicario@unict.it (N.V.); federica.spitale94@gmail.com (F.M.S.); alessandragiu94@gmail.com (M.A.S.G.); 2 Medical Scientist Training Program, The University of Alabama at Birmingham, Birmingham, AL 35294, USA; jdb82@uab.edu; 3 Division of Hematology, “A.O.U. Policlinico Vittorio Emanuele”, University of Catania, 95123 Catania, Italy; cesarinagiallongo@yahoo.it; 4 Department of Biomedical and Biotechnological Sciences, Section of Biochemistry, University of Catania, 95123 Catania, Italy; livolti@unict.it (G.L.V.); d.tibullo@unict.it (D.T.); 5 Lab of Synthetic and Systems Biology, Department of Drug Sciences, University of Catania, 95123 Catania, Italy; m.gulisano@unict.it; 6 Lab of Neurogenesis and Repair, Department of Drug Sciences, University of Catania, 95123 Catania, Italy; gpleanza@unict.it; 7 Lab of Neurophysiology, Department of Biomedical and Biotechnological Sciences, Section of Physiology, University of Catania, 95123 Catania, Italy

**Keywords:** neural stem cell (NSC), steroids, clobetasol, sonic hedgehog signaling (Shh), smoothened (Smo), Gli, stem cell proliferation

## Abstract

Sonic hedgehog (Shh) signaling is a key pathway within the central nervous system (CNS), during both development and adulthood, and its activation via the 7-transmembrane protein Smoothened (Smo) may promote neuroprotection and restoration during neurodegenerative disorders. Shh signaling may also be activated by selected glucocorticoids such as clobetasol, fluocinonide and fluticasone, which therefore act as Smo agonists and hold potential utility for regenerative medicine. However, despite its potential role in neurodegenerative diseases, the impact of Smo-modulation induced by these glucocorticoids on adult neural stem cells (NSCs) and the underlying signaling mechanisms are not yet fully elucidated. The aim of the present study was to evaluate the effects of Smo agonists (i.e., purmorphamine) and antagonists (i.e., cyclopamine) as well as of glucocorticoids (i.e., clobetasol, fluocinonide and fluticasone) on NSCs in terms of proliferation and clonal expansion. Purmorphamine treatment significantly increased NSC proliferation and clonal expansion via GLI-Kruppel family member 1 (Gli1) nuclear translocation and such effects were prevented by cyclopamine co-treatment. Clobetasol treatment exhibited an equivalent pharmacological effect. Moreover, cellular thermal shift assay suggested that clobetasol induces the canonical Smo-dependent activation of Shh signaling, as confirmed by Gli1 nuclear translocation and also by cyclopamine co-treatment, which abolished these effects. Finally, fluocinonide and fluticasone as well as control glucocorticoids (i.e., prednisone, corticosterone and dexamethasone) showed no significant effects on NSCs proliferation and clonal expansion. In conclusion, our data suggest that Shh may represent a druggable target system to drive neuroprotection and promote restorative therapies.

## 1. Introduction

Sonic hedgehog (Shh) is a signaling protein involved in both cell fate and differentiation [1], also playing a crucial role in both the development and maintenance of the adult central nervous system (CNS) [2]. Shh signaling involves a major receptor complex formed by the 12-transmembrane protein Patched (Ptch1) and one of its co-receptors, such as the cell adhesion molecule-related/down-regulated by oncogenes (Cdon) and biregional Cdon binding protein (Boc), which are anchored to the plasma membrane protein Gas-1 [3]. Once Shh binds to its receptor complex, the repression exerted by Ptch1 on the 7-transmembrane protein Smoothened (Smo) is alleviated, thereby resulting in its activation [1]; such signaling ultimately involves GLI-Kruppel family members 1 (Gli1) activation with Gli2 and Gli3 acting as transcriptional activators/repressors. This transduction pathway has been termed “canonical” and differs from “non-canonical” activation of Shh in that the latter does not involve Gli-mediated transcription and may also be Smo-independent (type I) or Smo-dependent (type II) [3]. Notably, dysregulation of the Shh pathway during development has been associated with a number of congenital anomalies and disorders [4,5,6]. Furthermore, aberrant Shh signaling has also been demonstrated to be involved in several brain pathologies, including cancer (e.g., medulloblastoma and glioma) [7,8] and CNS degenerative disorders (i.e., Parkinson’s disease and amyotrophic lateral sclerosis (ALS)) [9,10,11,12]. Other studies have also suggested a role for Shh in neurogenesis, angiogenesis and oligodendrogenesis in stroke [13] and in neuroinflammatory/demyelinating disorders [14,15]. Such findings are consistent with the role of Shh in controlling cell fate and proliferation of neural stem cells (NSCs) [16,17]. Accordingly, a number of studies have sought to therapeutically engage this key regulator of CNS disease/disorders [2,14,18,19]. In this regard, it has recently been shown that Food and Drug Administration (FDA)-approved glucocorticoids, including, clobetasol, fluocinonide and fluticasone, may also act as Smo agonists, thereby highlighting hitherto unrecognized potential in neuro-regenerative medicine [20,21,22,23]. In particular, clobetasol may induce remyelination via stimulation/differentiation of oligodendrocyte progenitor cells [24,25,26]. Critically, however, whether such activity is mediated by Smo agonism [23,26] and/or by binding to the glucocorticoid receptor Nuclear Receptor Subfamily 3 Group C Member 1 (NR3C1) [25] remains to be clarified. Therefore, the aim of the present study was to evaluate the effects of purmorphamine, a well-known Smo agonist [27], and selected FDA-approved glucocorticoids (i.e., clobetasol, flucinonide and fluticasone) on Shh pathway regulation in adult NSCs. Our results suggest that clobetasol activates canonical Shh signal activation targeting Smo, thus increasing NSCs growth and proliferation.

## 2. Results

### 2.1. Purmorphamine Increases the Growth Rate and Metabolism of NSCs

**Figure 1:**
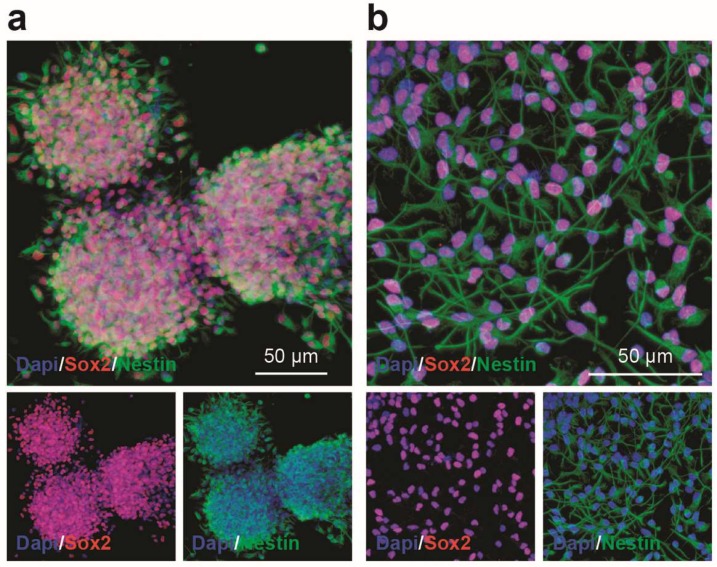
Mouse neural stem cells (NSCs) express Sox2 and Nestin, in vitro. (**a**,**b**) Representative confocal microscopy pictures of NSCs grown as neurospheres (**a**) and monolayer (**b**) expressing Sox2 (red) and Nestin (green). Nuclei were counterstained with Dapi (blue).

First, dosing of purmorphamine and cyclopamine was performed to assess both the potential cytotoxicity and the metabolic effects of these drugs on NSCs. Prior to treatments, cells were stained by immunofluorescence to assess the expression of NSC markers, thus showing that NSCs grown either as neurospheres or monolayer were Sox2 and Nestin positive (Figure 1a,b). 

**Figure 2:**
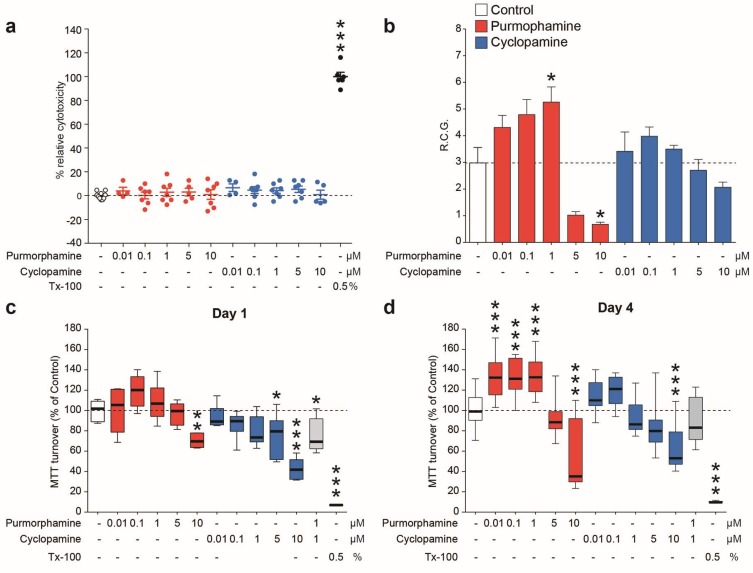
Purmorphamine activates sonic hedgehog (Shh) pathway within NSCs and increases the growth rate via activation of GLI-Kruppel family members 1 (Gli1). (**a**) Lactate dehydrogenase (LDH) viability assay on NSC cultures exposed to increasing concentration of purmorphamine and cyclopamine (0.01–10 μM). Data are shown as dots plot and viability is calculated as percentage of control assumed as 0%. (**b**–**d**) Dosing of purmorphamine and cyclopamine (from 0.01 to 10.0 μM) effects on NSCs performed by evaluating the rate of cell growth (RCG) (**b**) and the 3-(4,5-dimethylthiazol-2-Yl)-2,5-diphenyltetrazolium bromide (MTT) turnover of NSCs cultured for 24 h (**c**) and 4 days (**d**) in the presence of Shh pathway modulators. Data are mean numbers (± SEM) from n ≥ 3 independent experiments. * *p*-value < 0.05; ** *p*-value < 0.01; *** *p*-value < 0.001

The lactate dehydrogenase (LDH) viability assay showed that, at concentrations ranging from 0.01 to 10 μM, neither purmorphamine nor cyclopamine significantly increased cytotoxicity as compared to untreated cultures (Figure 2a), indicating that in our experimental conditions purmorphamine/cyclopamine exposition did not exert cytotoxic effects on NSCs. 

We then analyzed the effects of purmorphamine on the daily rate of cell growth (RCG) of NSCs; 1 μM concentration was able to increase the RCG by ~60% as compared to untreated control cultures (*p*-value < 0.05, Figure 2b), whereas higher doses (5 and 10 μM) led to a significant reduction of cell proliferation, reducing the NSCs daily RCG to ~1 (Figure 2b), effects that may be related to receptor or cell desensitization.

By contrast, treatment with cyclopamine did not significantly affect the RCG of NSCs at any of the assessed concentrations (Figure 2b). In order to investigate the metabolic turnover/integrity of mitochondria upon purmorphamine and/or cyclopamine treatment, a 3-(4,5-dimethylthiazol-2-Yl)-2,5-diphenyltetrazolium bromide (MTT) assay was performed after treating NSCs with drugs for either 1 (Figure 2c) or 4 (Figure 2d) days. 

**Figure 3:**
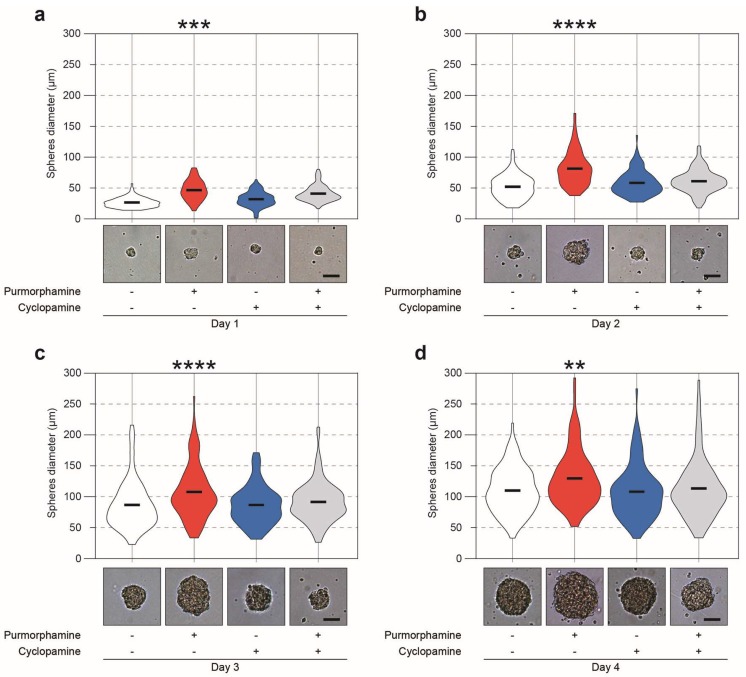
Shh pathway increases NSC neurosphere dimensions. (**a**–**d**) Violin plot of neurosphere diameters (expressed in μm) from day 1 (**a**), 2 (**b**), 3 (**c**) and 4 (**d**) in control (white) cultures and upon purmorphamine (red), cyclopamine (blue) or purmorphamine plus cyclopamine (gray) stimulation in vitro. Data from n ≥ 3 independent experiments. Scale bar 50 μm. ** *p*-value < 0.01, *** *p*-value < 0.001 and **** *p*-value < 0.0001 vs. day control.

Notably, a significant increase in MTT turnover appeared after 4 days of treatment with 0.01, 0.1 and 1 μM purmorphamine, as compared to untreated control cultures (132.1 ± 5.7% for 0.01 μM; 132.5 ± 4.8% for 0.1 μM and 133.6 ± 5.2% for 1 μM; 100.0 ± 4.5% for untreated cells; *p*-value < 0.001; Figure 2d). Importantly, 1 μM cyclopamine was able to revert the purmorphamine-induced increase of MTT turnover (89.2 ± 5.9%; *p*-value = 0.7236 vs. control cultures; Figure 2d). A significant decrease of MTT turnover below control levels was observed at the highest dose of both drugs (10 μM) after both 1 and 4 days of treatment (Figure 2c,d). Therefore, the 1 μM concentration of purmorphamine and cyclopamine was chosen for further experimentation. Using both purmorphamine and cyclopamine, we next sought to examine the effects of Shh pathway modulation on neurosphere diameter, as an index of clonal expansion and growth stimulation of NSCs. Neurospheres treated with purmorphamine were larger in diameter at every time-point (day 1: 49.4 ± 1.1 μm purmorphamine vs. 28.7 ± 0.7 μm control; day 2: 51.8 ± 1.8 μm purmorphamine vs. 79.2 ± 2.1 μm control; day 3: 87.7 ± 3.1 μm purmorphamine vs. 107.6 ± 3.5 μm control; day 4: 130.0 ± 3.9 μm purmorphamine vs. 120.3 ± 2.9 μm control; Figure 3a–d). This effect was abolished by cyclopamine (41.7 ± 0.8 μm, 57.8 ± 1.3 μm, 86.5 ± 2.1 μm and 103.5 ± 3.0 μm at days 1–4, respectively; Figure 3a–d). These data suggest the potential involvement of the Shh pathway in stimulating cell division, metabolic turnover and clonal expansion of NSCs.

### 2.2. Purmorphamine Activates “Canonical” Gli1-Dependent Shh Signaling in NSCs

**Figure 4:**
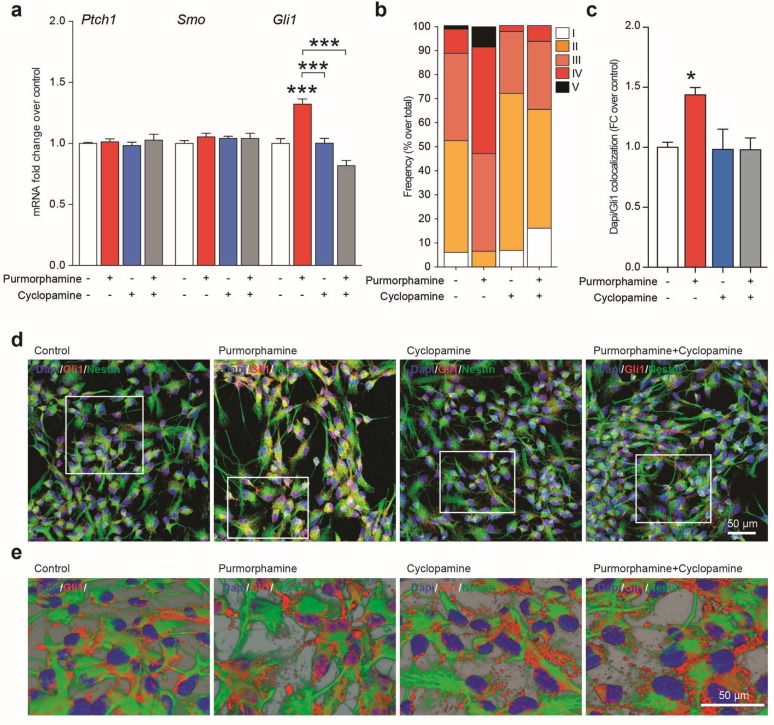
Purmorphamine induces Gli1 activation within NSCs. (**a**) mRNA levels of patched (Ptch1), smoothened (Smo) and Gli1 in control NSCs and NSCs exposed to 1 μM of purmorphamine, cyclopamine or purmorphamine plus cyclopamine. Data are mean fold change over control (±SEM) of n = 3 independent replicates. (**b**) Quantification of the intensity range (I and II = low; III = medium; IV and V = high) frequency of Dapi/Gli1 colocalization in control NSCs and NSCs exposed to 1 μM of purmorphamine, cyclopamine or purmorphamine plus cyclopamine. (**c**–**d**) Dapi/Gli1 colocalization (**c**) and representative confocal pictures (**d**) in control NSCs and NSCs exposed to 1 μM of purmorphamine, cyclopamine or purmorphamine plus cyclopamine. (**e**) 3D deconvolutions of Gli1 and Nestin of the confocal images in (**c**) highlighting the Dapi-Gli1 juxtaposition. * *p*-value < 0.05; *** *p*-value < 0.001.

To characterize the molecular dynamics altered/induced by purmorphamine/cyclopamine modulation of Shh signaling, we analyzed the mRNA levels of key mediators of hedgehog signaling. We found that neither purmorphamine and cyclopamine altered levels of the Shh receptor Ptch1 (Figure 4a). Surprisingly, mRNA levels of Smo (i.e., the main target of both purmorphamine and cyclopamine), were also unaffected by treatment with either compounds (Figure 4a). Next, we analyzed the mRNA levels of Gli1 in order to confirm the “canonical” Gli-dependent Shh activation supposedly induced by purmorphamine; we found that purmorphamine treatment increased Gli1 mRNA (Figure 4a), and that cyclopamine did not affect Gli1 levels; critically, cyclopamine cotreatment with purmorphamine abolished the previously noted Gli1 mRNA increase (Figure 4a). Given that Gli1 is a transcription factor, we sought to evaluate its localization upon Shh pathway activation. This set of experiments showed that Gli1 translocated to the nucleus upon exposure to purmorphamine with higher proportion of cells showing high Dapi-Gli1 colocalization (Figure 4b–e) and that cyclopamine co-treatment abolished such effect (Figure 4b–e). 

These data confirm that adult NSCs have functional/targetable Smo and that purmorphamine, a classical Smo agonist, activates “canonical” hedgehog signaling in adult NSCs, exerting its functional effects in part via Gli1-mediated signaling.

### 2.3. Clobetasol Increases Proliferation and Dimension of NSCs Neurospheres

**Figure 5:**
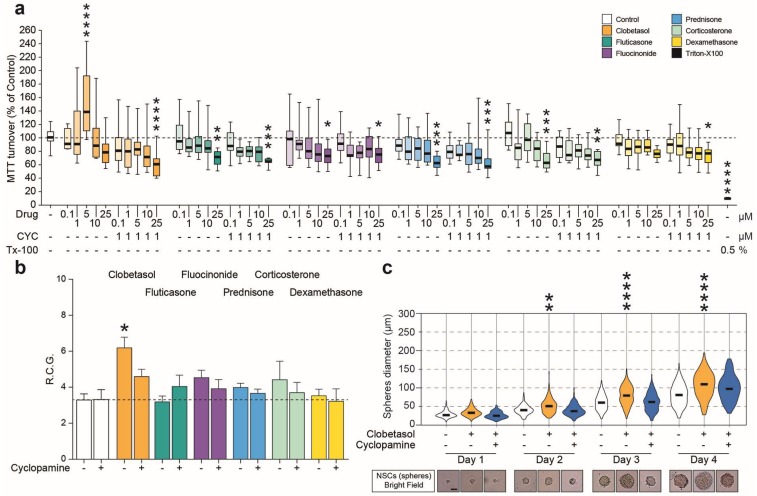
Clobetasol increases proliferation and neurosphere dimension. (**a**) MTT turnover on NSCs under treatment with glucocorticoids with potential activity on Shh pathway (Clobetasol, Fluticasone and Fluocinonide), and control glucocorticoids treatment (Prednisone, Corticosterone and Dexamethasone, all lacking Smo activity). Data are mean numbers (± SEM) from n ≥ 3 independent experiments. * *p*-value < 0.05, ** *p*-value < 0.01, *** *p*-value < 0.001 and **** *p*-value < 0.0001 vs. control. (**b**) NSCs rate of cell growth (RCG) after stimulation with glucocorticoids (5.0 μM). Cyclopamine was used to inhibit the effects on the Shh pathway. Data are mean numbers (± SEM) from n ≥ 3 independent experiments. ** *p*-value < 0.01 vs. untreated control. (**c**) Violin plot of the NSCs spheres diameter expressed in μm from day 1 to day 4 under Clobetasol treatment in vitro. Scale bar: 50 μm. Data from n ≥ 3 independent experiments. ** *p*-value < 0.01, **** *p*-value < 0.0001 vs. day control. Statistical analysis has been performed by using ANOVAs followed by Sidak post-hoc test.

In order to assess the translational relevance of the above findings, we next examined FDA-approved glucocorticoids for their ability to influence NSCs metabolism and cell growth; we analyzed the effects of clobetasol, fluticasone and fluocinonide, previously reported as potential activators of Shh signaling and prednisone, corticosterone and dexamethasone previously recognized as glucocorticoids which do not act on the hedgehog pathway. To evaluate the selective activation of Shh signaling via Smo, we employed 1 μM cyclopamine to antagonize the pathway and used concentrations, which ranged from 0.1 to 25 μM, of glucocorticoids. We found that all glucocorticoids mediated a dose-dependent reduction in MTT turnover (Figure 5a) with the exception of 5 μM clobetasol, which was able to increase the MTT turnover of NSCs (152.1 ± 17.9% 5 μM clobetasol vs. 101.3 ± 4.3% control; *p*-value < 0.0001; Figure 5a). These effects were reverted by cyclopamine, thereby indicating the potential involvement of Smo agonism and modulation of the Shh pathway. Then, the effects of clobetasol and of the other glucocorticoids (final concentration of 5 μM) on NSC growth were quantified, confirming that clobetasol, but not the other glucocorticoids, was in fact able to double the RCG of NSCs (Figure 5b), as well as to increase the neurosphere diameter (day 4: 100.0 ± 2.4 μm clobetasol vs. 79.5 ± 1.9 μm control; *p*-value < 0.0001; Figure 5c). Again, the effects mediated by clobetasol were selectively reverted by the Smo antagonist cyclopamine (Figure 5b,c).

### 2.4. Clobetasol Activates Canonical Shh Signaling by Smo Agonism and Gli1 Activation

**Figure 6:**
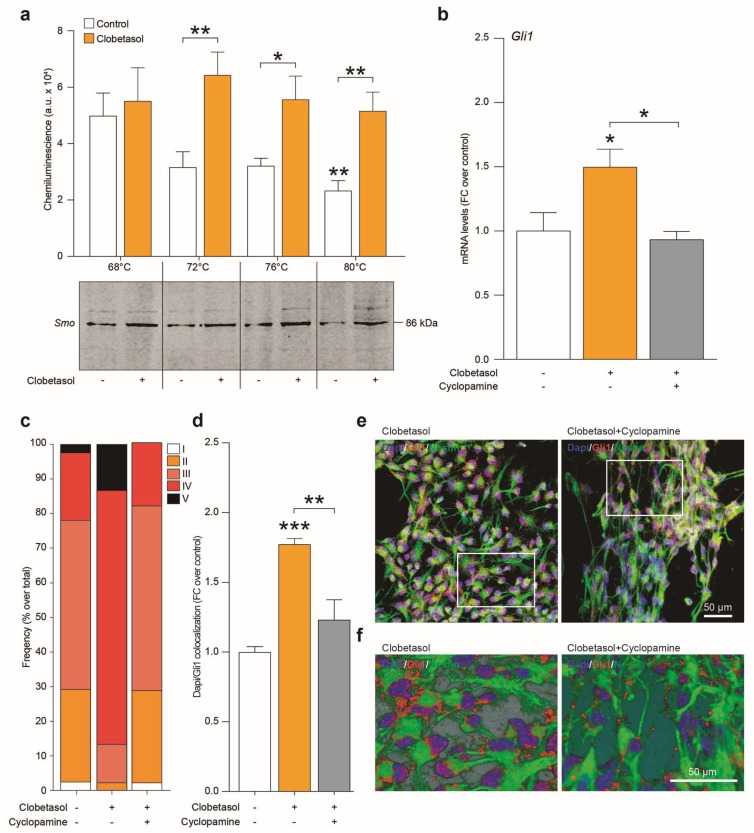
Clobetasol targets Smo activating canonical Shh signaling within NSCs. (**a**) Cellular thermal shift assay for target engagement with Smo. Clobetasol was subjected to cellular thermal shift assay (CETSA) at 4 different temperatures from 68 to 80 °C. Quantification and representative blot of target engagement. Data are means of n = 3 independent experiments. * *p*-value < 0.05, ** *p*-value < 0.01, *** *p*-value < 0.001 vs. control or between groups. (**b**) mRNA levels of Gli1 in NSCs exposed to clobetasol, cyclopamine or clobetasol plus cyclopamine. Data are mean fold change over control (±SEM) of n = 3 independent replicates. (**c**) Quantification of the intensity range (I and II = low; III = medium; IV and V = high) frequency of Dapi/Gli1 colocalization in control NSCs and NSCs exposed to clobetasol, cyclopamine or clobetasol plus cyclopamine. (**d**–**f**) Quantification of Dapi/Gli1 colocalization (d); representative confocal pictures (**e**) and 3D deconvolutions (**f**) of Gli1 and Nestin in control NSCs and NSCs exposed to clobetasol, cyclopamine or clobetasol plus cyclopamine.

The potential capability of clobetasol to selectively engage the Smo receptor was confirmed by Cellular thermal shift assay (CETSA). Critically, clobetasol demonstrated the ability to stabilize the Smo protein at a temperature above the melting point (*p*-value = 0.0087 for control 80 °C vs. control 68 °C; Figure 6a), indicating ligand-binding-induced stabilization (*p*-value > 0.9999 for clobetasol 80 °C vs. control 68 °C and *p*-value = 0.0051 for control 80 °C vs. clobetasol 80 °C; Figure 6a).

Our results showed that clobetasol treatment also significantly increased Gli1 mRNA levels (Figure 6b) and that this effect was selectively reverted by cyclopamine co-treatment (Figure 6b), thereby confirming that clobetasol acts as a Smo agonist activating “canonical” Gli1-dependent Shh signaling pathway. Finally, the quantification of Dapi/Gli1 colocalization in clobetasol-stimulated NSCs revealed an increased translocation of Gli1 upon clobetasol-induced Smo activation and a selective reduction of this phenomenon in NSCs co-treated with cyclopamine (Figure 6c–f).

## 3. Discussion

The Shh pathway is a key regulator of neurogenesis and cell fate during development/adulthood [2,4,28,29,30], and has been implicated in neuroprotective and compensatory mechanisms after CNS injuries or during neurodegenerative/neuroinflammatory disorders [10,13,14,31,32,33]. Moreover, the involvement of Shh signaling in spinal cord plasticity was also demonstrated in our laboratory using an in vivo mouse model of neurotoxic motoneuron degeneration induced by intramuscular injection of cholera toxin-B saporin [34,35,36,37,38]. In particular, these experiments demonstrated that mice are capable of locomotor recovery, possibly associated with the expression of Shh. However, whether these plastic changes can be stimulated via modulation of the Shh pathway, and whether they involve local activation/proliferation of NSCs is still unclear. In the present study, we sought to evaluate the effects of Shh pathway manipulation following the addition of Smo modulating agents in cultures of NSCs. These cells were isolated from mouse SVZ, cultured as neurospheres and characterized before treatments, thus showing Sox2 and Nestin expression. While an increasing body of evidence suggests a potential role for Smo agonists in neuroprotection and neuro-regeneration [10,25,32,36], the underlying molecular mechanisms have yet to be fully clarified. Within this work, the effects of well-known Smo modulators were assessed in NSCs in an effort to delineate how such Shh signaling perturbations would affect metabolic turnover and cell growth. 

These sets of experiments showed that the activation of the Shh pathway by purmorphamine strongly increased mitochondrial activity, RCG and clonal expansion of NSCs, effects that were abrogated by co-treatment with the known Smo antagonist cyclopamine. 

The use of these defined small molecules, together with the evaluation of key mediators of the Shh pathway, prompted us to assess whether the observed effects were exerted by the activation of either canonical or non-canonical Shh signaling [3,21]. Our data demonstrated that purmorphamine increased Gli1 translocation into the nucleus and that cyclopamine inhibited this effect, therefore suggesting that the observed NSC phenotypes are likely dependent on the activation of the canonical Shh pathway. It is also worth noting that, unlike Gli1 mRNA, which increased after stimulation, suggesting that Gli1 nuclear translocation exerts a positive loop on mRNA transcription at this time-point, we did not find significant modulation of either Ptch1 or Smo mRNA levels at 24 h post-stimulation, thus indicating that these mediators may have a different dynamic modulation as compared to the transcription factor Gli1. These results indicate that adult SVZ-derived NSCs retain both functional and druggable Shh; such findings may ultimately inform preclinical studies aimed at modulating endogenous NSC compartments and/or therapeutic NSC grafts in animal models of neurodegenerative diseases/disorders [10,25].

Given such a contention, it is clear that the development of novel Smo agonists and/or the repurposing/repositioning of currently approved drugs capable of modulating the Shh pathway are critically important, i.e., both from a basic science and clinical perspective [21,35,36]. This is an intriguing but poorly explored issue with potential impact in several CNS diseases/disorders, such as those related to the motoneurons, as suggested by the involvement of Shh signaling in functional recovery and plasticity in models of selective motoneuronal loss [35,36,38]. The present findings unravel novel mechanisms involved in Shh signaling modulation, thus offering further insights with therapeutic relevance in neurodegenerative diseases, including ALS therapy, which is currently based on glutamate toxicity and oxidative stress and is incapable of preventing progression of such a complex disease [39,40,41].

Another important issue is the availability of drugs acting on the Shh pathway, which are already approved and suitable for repurposing. Therefore, after confirming the effects of Smo agonism on NSCs by known Smo modulators (purmorphamine and cyclopamine), the present study tested the effects of clobetasol as a potential Shh pathway activator, demonstrating its ability to activate the canonical, Smo- and Gli1-dependent Shh signaling on NSCs. Consistent with these results, clobetasol was found to act as a mitogen throughout a Smo targeting in SVZ-derived NSCs. These effects not only depend on the stable engagement of Smo by clobetasol but may also be reverted by cyclopamine co-treatment. Clobetasol was selected among different FDA-approved glucocorticoids that are known to work also as Smo agonists, together with their known activity on glucocorticoid receptor [20,23,25,26]. In particular, fluocinonide and fluticasone were preliminarily screened, together with clobetasol, to evaluate their phenotypical effects on NSCs, thus showing that clobetasol exerted the most evident effects on MTT turnover, cell growth and clonal expansion. Therefore, modulating Shh signaling using Smo targeting compounds may represent a potential neuroprotective and regenerative strategy to foster self-repairing potential during neurodegenerative diseases.

## 4. Materials and Methods

### 4.1. Neural Stem Cell Derivation and Culture

Experiments were performed in accordance with the principles of the Basel Declaration as well as of the European Community Council Directive and Italian regulations (ECC Council 2010/63/EU and Italian D.Lgs. no. 26/2014). Moreover, the study was conducted in accordance with the recommendations of the local committee for animal welfare (OPBA, University of Catania, Via Santa Sofia 97, Catania, Italy); the protocol was approved by OPBA and by the Italian Ministry of Health (auth. no. 1133/2016-PR). All efforts were made to minimize animal suffering and to use the fewest animals possible. NSCs were derived from the subventricular zone (SVZ) of 8–12 weeks old (20–24 g) male 129/SV mice (Charles River Laboratories Italia s.r.l., Lecco, Italy) as has been previously described [42,43]. Briefly, mice were humanely culled by cervical dislocation and brains were isolated and washed with Earle’s balanced salt solution (EBSS, Sigma, Milan, Italy). Coronal 3 mm-thick blocks were collected and the SVZs were isolated and pooled together with samples from two additional animals. After a 45 min digestion at 37 °C with papain (1 mg/mL, Sigma), ethilenediaminotetraacetic acid (EDTA, 0.2 mg/mL, Sigma), L-cysteine (0.2 mg/mL, Sigma) in EBSS, the suspension was centrifuged (200× *g* for 12 min) and the pellet was mechanically triturated in EBSS. After an additional centrifugation, cells were seeded in NSC proliferation medium (EGF 20 ng/mL, bFGF 10 ng/mL, heparin 2 μg/mL, Sigma), mouse NeuroCult proliferation supplements (Stem Cell Technologies, Milan, Italy) in mouse NeuroCult basal medium (Stem Cell Technologies). After 6 days, isolated cells gave rise to neurospheres (100–150 μm diameter) that were collected in a tube and enzymatically digested with Accumax (Life Technologies, Milan, Italy) at 37 °C for 10 min. Viable cells were seeded at the clonal density of 10,000 cells/cm^2^. The RCG was calculated by counting the number of viable cells by vital stain exclusion (i.e., trypan blue staining) and dividing it by the number of plated cells; ratios were divided by the number of days per passage. All experiments employed cells at passage n < 20.

### 4.2. Shh Pathway Modulation in NSCs

The definitive modulation of the Shh signaling pathway was first achieved using a well-known Smo agonist (purmorphamine) and/or an antagonist (cyclopamine). Then, glucocorticoids with Smo activity (i.e., clobetasol, fluticasone, fluocinonide) and control glucocorticoids (i.e., prednisone, corticosterone, dexamethasone) were also employed. The following compounds were used at the indicated working dilutions: Purmorphamine (0.01–10 μM, Cat# 72204, Stem Cell Technologies), cyclopamine (0.01–10 μM, Cat# S1146, Selleckchem, Rome, Italy), clobetasol propionate (0.1–25 μM, Cat# C8037, Sigma), fluticasone propionate (0.1–25 μM, Cat# PHR1702, Sigma), fluocinonide (0.1–25 μM, Cat#SML0099, Sigma), prednisone (0.1–25 μM, Cat# P6254, Sigma), corticosterone (0.1–25 μM, Cat# 27840, Sigma) and dexamethasone (0.1–25 μM, Cat# D1756, Sigma). Drugs were diluted in cell culture media and cells were exposed to drugs and ultimately observed for 1–4 days.

### 4.3. LDH and MTT Cytotoxicity/Viability Assays

For LDH or MTT tests, neurospheres were dissociated and seeded in 96-well plates (Costar, Milan, Italy) at a final density of 30,000 cells/well/100 μL) and cultured for 1–4 days. After seeding, cells were exposed to drugs as above, and cultured using standard conditions. On the day of the test, medium was removed and processed as per manufacturer’s instructions for the LDH-viability assay (CytoSelectTM LDH cytotoxicity assay kit, Cell Biolabs, Milan, Italy). The MTT assay was performed as previously described [44], on separate plates. Briefly, a solution of MTT at a final concentration of 5 mg/mL was added to each well and incubated for 2 h at 37 °C/5% CO_2_. Media were then gently removed, MTT solvent (DMSO, Sigma) was added, and cells were stirred on an orbital shaker for 5 min at room temperature. The absorbance was measured using a Varioskan Flash spectrophotometer (Thermo Scientific, Milan, Italy) at 550 nm. Results were expressed as the percentage of MTT reduction versus control cells. Each experiment was performed three times with six replicates per condition during each experimental run.

### 4.4. Target Engagement Assay by CETSA and Immunoblot

CETSA was used to investigate target engagement of clobetasol, as previously described [45,46]. Cells were suspended in PBS at a final density of 5 × 10^6^ cells/mL, lysed via ultrasonication on ice, and treated with 5 μM of clobetasol propionate for 1 h at 37 °C. The cell suspension was divided in 4 aliquots of 100 μL and heated for 3.5 min at 68, 72, 76 or 80 °C, respectively. Samples were centrifuged at 15,000× *g* for 20 min at 4 °C to separate stable and denatured proteins, and supernatants were then collected and mixed with 4× Laemmli loading buffer and 10% β-mercaptoethanol and incubated at 95 °C for 5 min. Proteins were separated on 4–20% Tris-glycine acrylamide gels (Thermo Scientific) and transferred to nitrocellulose membranes. Membranes were incubated for 1 h at room temperature with Odyssey blocking buffer solution and then overnight at 4 °C with rabbit anti-Smo antibody (Abcam, Cat# ab72130, RRID: AB_1270802, 1:1000). After washes in 0.1% tween-20 in PBS, membranes were incubated for 1 h at room temperature with the secondary antibody (goat polyclonal anti-rabbit IRDye 680RD; LI-COR Biosciences, Cat# 926-68171, RRID: AB_10956389, 1:10,000). All antibodies were diluted in Odyssey blocking buffer solution. Proteins bands were imaged using an Odyssey Infrared Imaging Scanner (LI-COR Biosciences, Milan, Italy) and protein levels were quantified by densitometric analysis. Target engagement of clobetasol was quantitatively evaluated as a significant increase in remaining Smo protein at temperatures that were lower than, equal to or higher than the melting temperature, in comparison to control samples treated with vehicle (DMSO) alone.

### 4.5. Immunofluorescence

For immunofluorescence, paraformaldehyde (PFA)-fixed cells samples were permeabilized in 0.1% Triton X100 in PBS and incubated with blocking solution (10% normal goat serum, NGS, in 0.1% Triton X100 in PBS) for 1 h at room temperature [47]. Samples were then incubated overnight at 4 °C with the following primary antibodies diluted in incubating solution (1% NGS in 0.1% Triton X100 in PBS): Rabbit anti-Gli1 (Abcam, Cat# ab49314, RRID: AB_880198, 1:1000), chicken anti-Nestin (Abcam, Cat# ab134017, RRID: AB_2753197, 1:500) and rabbit anti-Sox2 (Abcam, Cat# ab97959, RRID: AB_2341193, 1:1000). Then, after 3 washes with 0.1% Triton X100 in PBS, samples were incubated for 1 h at room temperature with the appropriate fluorescence goat secondary antibodies: Anti-rabbit 546 (Invitrogen, Cat# A11010, RRID: AB_143156, 1:1000) and anti-chicken 488 (Abcam, Cat# ab150169, RRID: AB_2636803, 1:1000). Nuclei were counterstained with 4′,6-diamidino-2-phenylindole (Dapi, 1:1000, Cat# D1306, Invitrogen) for 5 min at room temperature. Slides were mounted with fluorescent mounting medium Permafluor (ThermoScientific) and digital images were acquired using a Leica DM IRB (Leica Microsystems, Buccinasco, Milano, Italy) fluorescence microscope or the Leica TCS SP8 confocal microscope. For quantification of Gli1 and Dapi colocalization, NSCs somata (Dapi) were segmented and the corresponding Gli1 fluorescence intensity was quantified using ImageJ analysis software and the frequency of low (I and II), medium (III) and high (IV and V) nuclear Gli1 fluorescence intensity was obtained as percentage over total nuclei. The overall intensity of Dapi/Gli1 colocalization was quantified by ImageJ (v. 1.52d, NIH, Bethesda, MD, USA) colocalization threshold and expressed as fold change over control NSCs.

### 4.6. mRNA Quantification

The QuantiGene Plex Magnetic Separation Assay kit was used (Affymetrix, Santa Clara, CA, USA). All experiments were performed according to manufacturer’s instructions. Briefly, 500 ng of RNA was added to separate wells of a 96-well hybridization plate containing magnetic capture beads and QG_2.0 probe sets. The plate was incubated for 22 h at 55 °C and agitated at 600 rpm to maintain the suspension. Beads were sequentially hybridized (1 h, 50 °C at 600 rpm) with preamplifier probe, the amplifier probe, the label probe and phycoerythrin-conjugated streptavidin, with 3 washes in washing buffer in between, followed by a final 30-min incubation at 37 °C with developing solution. Bead discrimination and signal detection were performed on a Luminex instrument (Bio-Rad, Milan, Italy). For each sample the average signal (MFI) for Ptch1, Smo, Gli1 and actb were determined and, after average background signal subtraction, each gene signal (background subtracted) was divided by the normalization gene signal (background subtracted): actb. Data are shown as mean (± SEM) fold change over control.

### 4.7. Statistical Analyses

All statistics were performed using GraphPad Prism (version 5.00 for Mac, GraphPad Software, San Diego, CA, USA) or RStudio (version 1.0.153, RStudio Inc., Boston, MA, USA). Data were tested for normality using a D’Agostino and Pearson omnibus normality test and subsequently assessed for homogeneity of variance. Data that passed both tests were further analyzed by two-tailed unpaired Student’s t-test for comparison of n = 2 groups. Comparisons of n > 2 groups were performed using a one-way ANOVA and Holm-Sidak’s multiple comparisons test. For all statistical tests, *p*-values < 0.05 were considered statistically significant; *p*-values are reported within the figure legends.

## 5. Conclusions

In conclusion, we have demonstrated that by engaging Smo-dependent signaling with purmorphamine or by the FDA-approved glucocorticoid clobetasol, the hedgehog pathway was modulated on NSCs and induced effects that are related to the canonical Shh signaling pathway, such as proliferation and cell metabolism. Taken together, this evidence suggests that the Shh pathway may play a crucial role in neurodegenerative diseases, and it may also represent a druggable system to be targeted in order to drive neuroprotection and potentiate repairing mechanisms. In vivo experiments are ongoing to confirm this hypothesis.

## Abbreviations

ALS	Amyotrophic lateral sclerosis
Boc	Biregional Cdon binding protein
Cdon	Cell adhesion molecule-related/down-regulated by oncogenes
CETSA	Cellular thermal shift assay
CNS	Central nervous system
EBSS	Earle’s balanced salt solution
EDTA	Ethilenediaminotetraacetic acid
FDA	Food and Drug Administration
Gli1	GLI-Kruppel family members 1
LDH	Lactate dehydrogenase
MTT	3-(4,5-dimethylthiazol-2-Yl)-2,5-diphenyltetrazolium bromide
NGS	Normal goat serum
NR3C1	Nuclear Receptor Subfamily 3 Group C Member 1
NSCs	Neural stem cells
PFA	Paraformaldehyde
Ptch1	Patched
RCG	Rate of cell growth
SEM	Standard error of the mean
Shh	Sonic hedgehog
Smo	Smoothened
SVZ	Subventricular zone
